# Aucubin Exerts Neuroprotection against Forebrain Ischemia and Reperfusion Injury in Gerbils through Antioxidative and Neurotrophic Effects

**DOI:** 10.3390/antiox12051082

**Published:** 2023-05-11

**Authors:** Joon Ha Park, Tae-Kyeong Lee, Dae Won Kim, Ji Hyeon Ahn, Choong-Hyun Lee, Soon Sung Lim, Yang Hee Kim, Jun Hwi Cho, Il Jun Kang, Moo-Ho Won

**Affiliations:** 1 Department of Anatomy, College of Korean Medicine, Dongguk University, Gyeongju 38066, Republic of Korea; 2 Department of Food Science and Nutrition, Hallym University, Chuncheon 24252, Republic of Korea; 3 Department of Biochemistry and Molecular Biology, Research Institute of Oral Sciences, College of Dentistry, Gangnung-Wonju National University, Gangneung 25457, Republic of Korea; 4 Department of Physical Therapy, College of Health Science, Youngsan University, Yangsan 50510, Republic of Korea; 5 Department of Pharmacy, College of Pharmacy, Dankook University, Cheonan 31116, Republic of Korea; 6 Department of Surgery, Kangwon National University Hospital, School of Medicine, Kangwon National University, Chuncheon 24289, Republic of Korea; 7 Department of Emergency Medicine, Kangwon National University Hospital, School of Medicine, Kangwon National University, Chuncheon 24289, Republic of Korea

**Keywords:** aucubin, hippocampus, histopathology, immunohistochemistry, oxidative stress, transient ischemia, western blotting

## Abstract

Aucubin is an iridoid glycoside that displays various pharmacological actions including antioxidant activity. However, there are few reports available on the neuroprotective effects of aucubin against ischemic brain injury. Thus, the aim of this study was to investigate whether aucubin protected against damage to hippocampal function induced by forebrain ischemia-reperfusion injury (fIRI) in gerbils, and to examine whether aucubin produced neuroprotection in the hippocampus against fIRI and to explore its mechanisms by histopathology, immunohistochemistry, and Western analysis. Gerbils were given intraperitoneal injections of aucubin at doses of 1, 5, and 10 mg/kg, respectively, once a day for seven days before fIRI. As assessed by the passive avoidance test, short-term memory function following fIRI significantly declined, whereas the decline in short-term memory function due to fIRI was ameliorated by pretreatment with 10 mg/kg, but not 1 or 5 mg/kg, of aucubin. Most of the pyramidal cells (principal cells) of the hippocampus died in the Cornu Ammonis 1 (CA1) area four days after fIRI. Treatment with 10 mg/kg, but not 1 or 5 mg/kg, of aucubin protected the pyramidal cells from IRI. The treatment with 10 mg/kg of aucubin significantly reduced IRI-induced superoxide anion production, oxidative DNA damage, and lipid peroxidation in the CA1 pyramidal cells. In addition, the aucubin treatment significantly increased the expressions of superoxide dismutases (SOD1 and SOD2) in the pyramidal cells before and after fIRI. Furthermore, the aucubin treatment significantly enhanced the protein expression levels of neurotrophic factors, such as brain-derived neurotrophic factor and insulin-like growth factor-I, in the hippocampal CA1 area before and after IRI. Collectively, in this experiment, pretreatment with aucubin protected CA1 pyramidal cells from forebrain IRI by attenuating oxidative stress and increasing neurotrophic factors. Thus, pretreatment with aucubin can be a promising candidate for preventing brain IRI.

## 1. Introduction

Transient global brain ischemia occurs in a multitude of clinical situations, including cardiac arrest [1] and severe hypotension and shock [2], and eventually leads to irreversible neuronal damage in several brain areas such as the hippocampal formation (hippocampus) and cerebral cortex [3,4]. Especially among the subfields of the hippocampus, the CA1 area is the most vulnerable to forebrain ischemia and reperfusion injury (fIRI) [5], showing that selective death (loss) of principal cells (pyramidal cells or neurons) in the CA1 area occurs several days after fIRI [6,7,8]. Such a phenomenon is commonly referred to as “delayed neuronal death (DND)” [6]. The complex mechanisms of brain tissue damage including DND have been widely studied and proposed in experimental animal models [9].

One of the crucial pathophysiological causes of brain tissue damage following ischemic insults is oxidative stress [10,11], which refers to the pathological process of brain tissue damage caused by the overproduction of reactive oxygen species (ROS) or reduction of its scavenging capacity during metabolic processes and results in an imbalance between antioxidant and oxidative systems [12]. Oxidative stress occurs when an imbalance between antioxidants and free radicals, including ROS, shows that DNA, lipids, and proteins are damaged [13]. In brain ischemic insults, oxidative stress damages the oxidation/antioxidation balance shows that neuronal mitochondria produce a large amount of ROS and hydroxyl radicals and lead to neuron death [12,14]. In light of this, antioxidant strategies to reduce oxidative stress may be one of the effective approaches for the attenuation of fIRI-induced DND.

Aucubin is an iridoid glycoside and has been used as an active constituent of traditional medicinal plants including *Veronica persica* and *Plantago lanceolata* L. [15,16]. It has been reported that aucubin has multiple pharmacological actions including antioxidant and anti-inflammatory activities [17,18]. Especially, it has been reported that aucubin displays a pancreas-protective effect via its antioxidant action in a rat model of streptozotocin-induced diabetes, showing that aucubin mitigates the level of lipid peroxidation and improves antioxidant enzymes [17]. In addition, aucubin inhibits hydrogen peroxide-induced apoptosis through regulating the endogenous oxidant-antioxidant balance in adrenal medulla pheochromocytoma (PC12) cells [19]. Due to these actions, aucubin has been used medicinally for the treatment and management of neurological disorders (i.e., Parkinson’s disease) [20], diabetic encephalopathy [21], and traumatic brain injury [22]. However, to the best of our current knowledge, potential neuroprotective effects of aucubin against fIRI have not been demonstrated yet, although there are reports showing that aucubin displays protective effects against liver and heart ischemia-reperfusion injuries through its antioxidant activity in experimental rodent models [23,24]. Therefore, in this study, we evaluated the neuroprotective effects of aucubin and whether its effects occurred through antioxidant action in a gerbil model of fIRI which has been extensively used for assessing the efficacy and mechanisms of potential neuroprotective candidates [11,25].

## 2. Materials and Methods

### 2.1. Animals and Experimental Protocol

Healthy six-month-old male gerbils (70~80 g) were supplied by the Experimental Animal Center of Kangwon National University. The temperature of the feeding environment was controlled at 22 ± 2 °C, and the gerbils were housed on a 12-h light/dark cycle. The care and handling of the gerbils were conducted in accordance with the guidelines for the care and use of laboratory animals. All experimental procedures were approved (approval no., KW-200113-1) by the ethics committee of the Kangwon National University on 18 February 2020. During the experiments, pain in the gerbils was minimized.

### 2.2. Experimental Groups and Administration of Aucubin

First, to evaluate the dosage of aucubin to show neuroprotective effects against fIRI on day 4 after fIRI, 40 gerbils were used for (1) vehicle (saline)-sham group (*n* = 5) treated with vehicle and undergone sham fIRI operation; (2) vehicle-fIRI group (*n* = 5) treated with vehicle and undergone fIRI operation; (3), (4), and (5) were the 1, 5, and 10 mg/kg aucubin-sham group (*n* = 5 in each group) treated with 1, 5, and 10 mg/kg of aucubin, respectively, which had undergone sham operation; (6), (7), and (8) were the 1, 5, and 10 mg/kg aucubin-fIRI groups (*n* = 5 in each group) treated with 1, 5, and 10 mg/kg of aucubin, respectively, which had undergone fIRI operation. The five gerbils in each group were sacrificed on day 4 after fIRI because neuronal death occurs in the hippocampal CA1 area at this point in time [7]. In this experiment, we found that 10 mg/kg of aucubin displayed neuroprotection in the ischemic CA1 area.

Next, to examine the neuroprotective mechanisms of 10 mg/kg of aucubin, immunohistochemistry and Western blot analyses were conducted on day 1 after fIRI in the vehicle-sham group (*n* = 14), the vehicle-fIRI group (*n* = 14), the aucubin (10 mg/kg)-sham group (*n* = 14), and the aucubin (10 mg/kg)-fIRI group (*n* = 14). Eventually, 24 gerbils in each group were sacrificed for histological/immunohistochemical (*n* = 7, respectively) and Western blot (*n* = 5, respectively) analyses at 1 and 4 days after fIRI.

**Figure 1:**
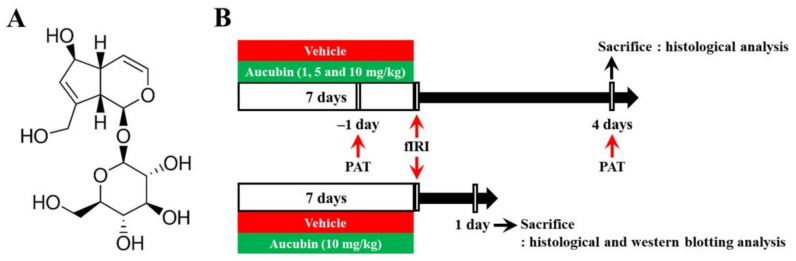
(**A**) The chemical structure of aucubin and (**B**) schematic representation of the experimental procedure. Aucubin was dissolved in saline and intraperitoneally injected once/day for 7 days before fIRI. Passive avoidance test (PAT) was conducted 1 day before and 4 days after fIRI. The gerbils were sacrificed to examine neuroprotection on day 1 and to investigate the mechanisms of the neuroprotection 4 days after fIRI.

Aucubin (purity, ≥98%; Sigma-Aldrich, St. Louis, MO, USA) was dissolved in saline as a vehicle. Aucubin or vehicle was intraperitoneally administered once every 24 h for a total of 7 consecutive days before fIRI surgery. The dose levels of aucubin were chosen according to the results of a recent study showing the protective effects of aucubin against liver ischemia-reperfusion injury in rats [24]. The chemical structure of aucubin and the experimental procedure are shown in Figure 1.

### 2.3. Induction of fIRI

fIRI was developed by occlusion of both common carotid arteries, as described in our published paper [7]. Briefly, the gerbils were anesthetized with 2.5 isoflurane (JW Pharmaceutical Corporation, Seoul, Republic of Korea) in 70/30% nitrous oxide/oxygen. Under the anesthesia, both (right and left) common carotid arteries were separated carefully in the ventral surface of the neck and ligated with non-traumatic aneurysm clips obtained from Fine Science Tools (Foster City, CA, USA) for five minutes. The complete blockage of blood supply during the occlusion was confirmed through observation of the central retinal artery using HEINE K180 ophthalmoscope (Heine Optotechnik, Herrsching, Germany). The clips were removed after five minutes of occlusion, and the operated neck incision was sutured. For the control of body temperature, rectal temperature was carefully monitored and controlled at normothermia (37 ± 0.5 °C) throughout the surgery using heating pads (Harvard Apparatus, Holliston, MA, USA). After recovery from the surgery, the gerbils were returned to their home cages. For the sham-operated gerbils in this experiment, the gerbils underwent identical surgery except clips were not applied.

### 2.4. Passive Avoidance Test (PAT)

As described previously [26], PAT was conducted with minor modifications. In short, we used Gemini Avoidance System (GEM 392, San Diego Instruments Inc., San Diego, CA, USA) consisting of dark and light compartments, which are separated by a vertical sliding gate. The test was performed with two sessions, as training and substantial trials. The training session was done one day before fIRI. For training, each gerbil was placed in the light compartment and allowed to explore the inner structure of the compartment for one minute. When the gerbil entered the dark compartment, the sliding gate was closed, and the gerbil received an electric foot shock (0.5 mA) for five seconds from the iron-grid floor. On day 4 after fIRI, the substantial trial was conducted. Individual gerbil was placed in the light compartment, and the latency time was recorded till the gerbil entered the dark compartment within 180 s.

### 2.5. Tissue Preparation for Histological Examination

All gerbils were deeply anesthetized with an intraperitoneal injection of urethane (1.5 g/kg; Sigma-Aldrich). Under deep anesthesia, the gerbils were transcardially rinsed with phosphate-buffered saline (PBS, 0.05 M, pH 7.4) and fixed with 4% paraformaldehyde (Shamchun Chemical Co., Ltd., Seoul, Republic of Korea). Thereafter, their brains were eliminated from the skulls and received more fixative for eight hours. For the preparation of brain sections, the brains were soaked in 30% sucrose for cryoprotection when they were cut. The brains were coronally sectioned into 25-micrometer thick slices on a freezing stage sliding microtome (Leica Microsystems, Wetzlar, Germany).

### 2.6. Nissl Staining

Nissl staining (a method to study the pathology of neurons) was carried out using cresyl violet (CV) dye (Sigma-Aldrich), as previously described [11]. Briefly, the sections were transferred to 0.1% CV solution for 20 min at room temperature, stained with CV, quickly rinsed with distilled water, dehydrated in gradient alcohol, and transparented in xylene. Finally, they were cover-slipped with Canada balsam (Sigma-Aldrich) and observed under BX53 light microscope (Olympus, Tokyo, Japan).

### 2.7. Fluoro-Jade B (FJB) Fluorescence Staining

For the examination of fIRI-induced neuronal loss, histofluorescence staining with FJB (a high-affinity fluorescent marker for the detection of degenerating apoptotic and necrotic neurons) was conducted. As previously described [11], in short, the brain sections were immersed in 0.06% potassium permanganate for 15 min and then transferred to 0.0004% FJB (Histochem, Jefferson, AR, USA) for 20 min. Thereafter, they were briefly washed and immersed in xylene. Finally, they were cover-slipped with fluorescent mounting medium (Dako, Glostrup, Denmark).

For the quantification of FJB-stained neurons, referring to the gerbil brain atlas (between anterior −1.4 mm and posterior −2.2 mm from the bregma) [27], five sections per gerbil were examined. As previously described [11], in brief, the images of the FJB-stained neurons were captured in the hippocampal CA1 area using BX53 light/fluorescence microscope (Olympus) equipped with a DP-72 digital camera (Olympus). The FJB-stained neurons were counted and evaluated by averaging the numbers using computerized image analysis system (Optimas 6.5 software, CyberMetrics, Scottsdale, AZ, USA). The number of FJB-stained neurons was counted in 300 × 300 μm at the center in the CA1 region.

### 2.8. Dihydroethidium (DHE) Fluorescence Staining

ROS production in ischemic hippocampal CA1 area was examined using fluorescence staining with DHE, a fluorescent dye to monitor superoxide production (Sigma-Aldrich). As previously described [28], briefly, the fixed brain sections were incubated with 10 μmol/L fluorescent dye DHE in a light-protected humidified chamber for 30 min at 37 °C. Thereafter, the sections were cover-slipped and examined using a fluorescence microscope.

The fluorescence intensity of DHE was assessed at excitation wavelength of 520–540 nm based on our published method [28]. Briefly, red precipitations that reflect intracellular superoxide production in the hippocampal CA1 area were captured using BX53 fluorescence microscope. The images of the DHE-stained cells were captured. Finally, the fluorescence intensity was evaluated using Image-pro Plus 6.0 software obtained from Cybernetics Inc. (Silver Spring, MD, USA): a ratio of the fluorescence intensity was calibrated as % (vehicle-sham group, 100%).

### 2.9. Immunohistochemistry

Immunohistochemistry was conducted for fIRI-induced neuronal change, DNA damage, lipid peroxidation, and changes of endogenous antioxidant enzymes in the ischemic hippocampal CA1 area. In short, according to our published procedure [29], the sections were reacted with 3% H_2_O_2_ for 20 min to block the activity of endogenous peroxidase. The sections were briefly washed and incubated in corresponding primary antibody at 4 °C for 8 h. The primary antibodies were mouse anti-neuronal nuclei (NeuN; diluted 1:1000; Chemicon, Temecula, CA, USA), goat anti-8-hydroxydeoxyguanosine (8OHdG; diluted 1:500; Millipore, Billerica, MA, USA), mouse anti-4-hydroxy-2-nonenal (4HNE; diluted 1:800; Alexis Biochemicals, San Diego, CA, USA), sheep anti-Cu, Zn-superoxide dismutase (SOD1; diluted 1:1000, Calbiochem, La Jolla, CA, USA), and sheep anti-Mn-superoxide dismutase (SOD2; diluted 1:1000; Calbiochem). Thereafter, the sections were briefly washed, incubated in corresponding biotinylated secondary antibody (diluted 1:250; Vector Laboratories, Burlingame, CA, USA) at room temperature for 2 h and immersed in avidin–biotin complex solution (diluted 1:250; Vector Laboratories) at room temperature for 1 h. They were briefly washed and reacted in 0.05% 3,3′-diaminobenzidine tetrahydrochloride (Sigma-Aldrich) for visualization. Finally, they were mounted onto gelatin-coated slides and sealed with Canada balsam (Sigma-Aldrich).

For the quantification of NeuN-immunostained neurons, five sections per gerbil were examined in the same way as the evaluation of FJB-stained neurons. The numbers of NeuN-immunostained neurons were counted in 300 × 300 μm at the center of the CA1 region. To quantitatively analyze the changes of 8OHdG-, 4HNE-, SOD1-, and SOD2-immunoreactive structure, the digital image of each immunoreactive structure was obtained using BX53 light microscope [28]. The obtained image was converted into 16-bit grayscale images with a range from 0 (black) to 255 (white) and evaluated as relative optical density (ROD) using Adobe Photoshop 8.0 (San Jose, CA, USA) and Image J software (version 1.59) of the National Institutes of Health (Bethesda, MD, USA). ROD was relatively presented as % compared to the vehicle-sham group (100%).

### 2.10. Western Blot Analysis

Western blotting was conducted to examine changes in the levels of brain-derived neurotrophic factor (BDNF) and insulin-like growth factor-I (IGF-I) in the ischemic CA1 area. In short, according to a published method [30], CA1 tissues were dissected, collected, and lysed in the protein lysis buffer—50 mM Tris-HCI (pH 7.5) containing 150 mM NaCl, 1 mM phenylmethylsulfonyl fluoride, 0.5% deoxycholic acid, 0.1% sodium dodecyl sulfate, 1% nonidet-P40, and 100 μm/mL leupeptin. BDNF and IGF-I Protein concentrations were measured using a colorimetric protein assay kit obtained from Bio-Rad (Hercules, CA, USA) as follows. Protein samples of 40 μg were run on sodium dodecyl sulfate-polyacrylamide gel and transferred to nitrocellulose membranes (Schleicher and Schuell GmbH, Dassel, Germany). Thereafter, the membranes were incubated in 5% skim milk at 4 °C for 8 h and reacted with primary antibodies: rabbit anti-BDNF (diluted 1:1000; Abcam, Cambridge, MA, USA), rabbit anti-IGF-I (diluted 1:500; Santa Cruz Biotechnology, CA, USA), and rabbit anti-β-actin (diluted 1:5000; Sigma-Aldrich). Subsequently, they were exposed to peroxidase-conjugated rabbit anti-rabbit Immunoglobulin G (diluted 1:2000; Santa Cruz Biotechnology) for 1 h. Finally, band detection was carried out using an enhanced chemiluminescence detection kit obtained from Santa Cruz Biotechnology.

To compare the expressions of BDNF and IGF-I, the quantification of the protein bands was performed according to our published procedure [30]. In brief, the detected bands were scanned and densitometrically calculated using Scion Image software obtained from Scion Corp. (Frederick, MD, USA). The protein expressions were normalized by corresponding expression of β-actin.

### 2.11. Statistical Analysis

Data obtained in this study were shown as means ± standard errors of the mean (SEM). All of the statistical analyses were done using GraphPad Prism (version 5.0) obtained from GraphPad Software Inc. (La Jolla, CA, USA). The analysis of variance (ANOVA) with post hoc Bonferroni’s multiple comparison tests was conducted to determine differences between all experimental groups. Statistical significance was accepted for *p* values of <0.05.

## 3. Results

### 3.1. Protection of fIRI-Induced Decline in Short-Term Memory Function by Aucubin

**Figure 2:**
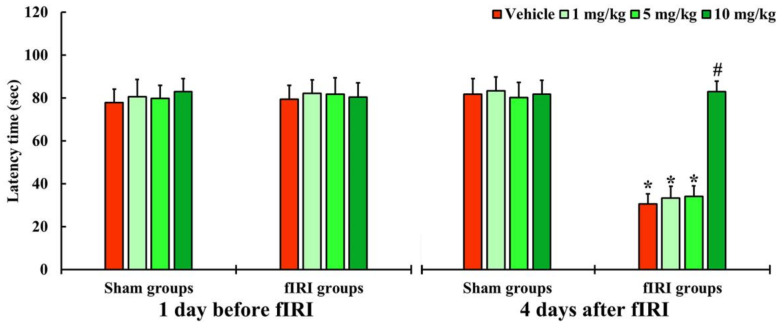
Latency time by PAT in the vehicle-sham, vehicle-fIRI, 1 mg/kg aucubin-sham, 1 mg/kg aucubin-fIRI, 5 mg/kg aucubin-sham, 5 mg/kg aucubin-fIRI, 10 mg/kg aucubin-sham, and 10 mg/kg aucubin-fIRI groups 1 day before and 4 days after fIRI. In the vehicle-fIRI, 1 and 5 mg/kg aucubin-fIRI groups, the latency times are significantly shortened when compared with the vehicle-sham group. On the other hand, in the 10 mg/kg aucubin-fIRI group, the latency time is similar to that evaluated in the sham-vehicle group. Values are expressed in mean ± SEM (*n* = 7, respectively; * *p* < 0.05 vs. each sham group, *# p* < 0.05 vs. vehicle-fIRI group).

In all groups, one day before fIRI, the latency times were not significantly different, indicating that the gerbils were subjected to identical pre-training (Figure 2). In the vehicle-fIRI, 1 and 5 mg/kg aucubin-fIRI groups, the latency times were significantly shortened at 4 days after fIRI when compared with those assessed in the vehicle-sham group showing that the short-term memory function declined following fIRI (Figure 2). On the other hand, the latency time in the 10 mg/kg aucubin-fIRI group was similar to that evaluated in the sham groups (Figure 2).

### 3.2. Neuroprotection of Aucubin against fIRI

#### 3.2.1. Findings by Nissl Staining

**Figure 3:**
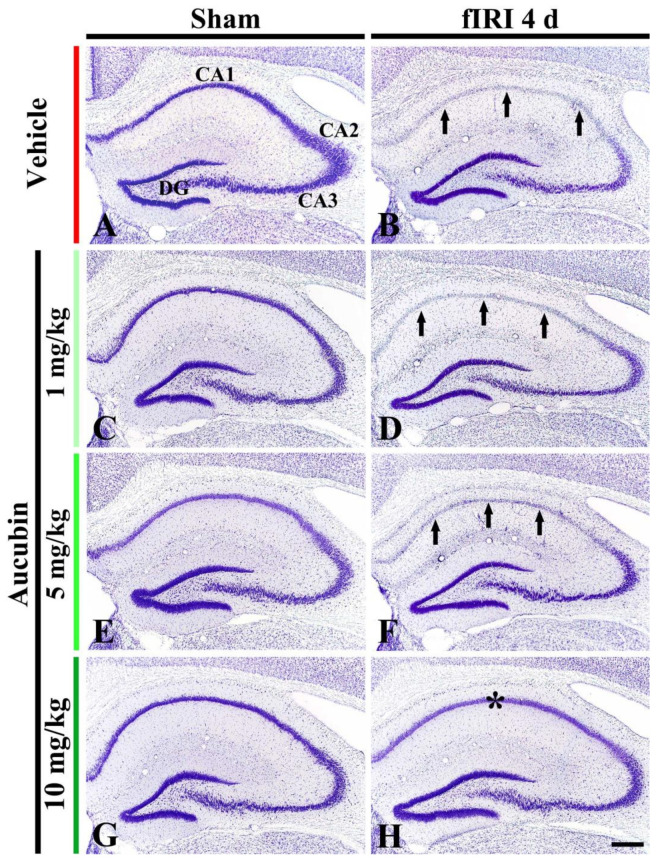
Representative images of Nissl staining in the hippocampus of the vehicle-sham (**A**), vehicle-fIRI (**B**), 1 mg/kg aucubin-sham (**C**), 1 mg/kg aucubin-fIRI (**D**), 5 mg/kg aucubin-sham (**E**), 5 mg/kg aucubin-fIRI (**F**), 10 mg/kg aucubin-sham (**G**), and 10 mg/kg aucubin-fIRI (**H**) groups at 4 days after fIRI. Note that CV stainability in the stratum pyramidale (arrows in (**B**,**D**,**F**)) of the CA1 area in the vehicle-fIRI, 1 mg/kg aucubin-fIRI, and 5 mg/kg aucubin-fIRI groups is pale after fIRI. However, in the 10 mg/kg aucubin fIRI group, strong CV stainability is shown in the CA1 stratum pyramidale (asterisk in (**H**)). CA, cornu ammonis; DG, dentate gyrus. Scale bar = 200 μm.

In the vehicle-sham group, CV stainability was easily distinguished in cells located in all subareas (CA1-3 area and dentate gyrus) of the hippocampus (Figure 3A). In the 1, 5, and 10 mg/kg aucubin-sham groups, CV stainability was not different from that shown in the vehicle-sham group (Figure 3C,E,G).

In the vehicle-fIRI group, very weak CV stainability was shown in cells located at the stratum pyramidale (called pyramidal neurons) of the CA1 area at 4 days after fIRI (Figure 3B). In the 1 and 5 mg/kg aucubin-fIRI groups, CV stainability in the CA1 pyramidal neurons was similar to that found in the vehicle-fIRI group (Figure 3D,F). However, in the 10 mg/kg aucubin-fIRI group, CV stainability in the CA1 pyramidal neurons was similar to that shown in the vehicle-sham group (Figure 3H).

#### 3.2.2. Findings by NeuN Immunohistochemistry

**Figure 4:**
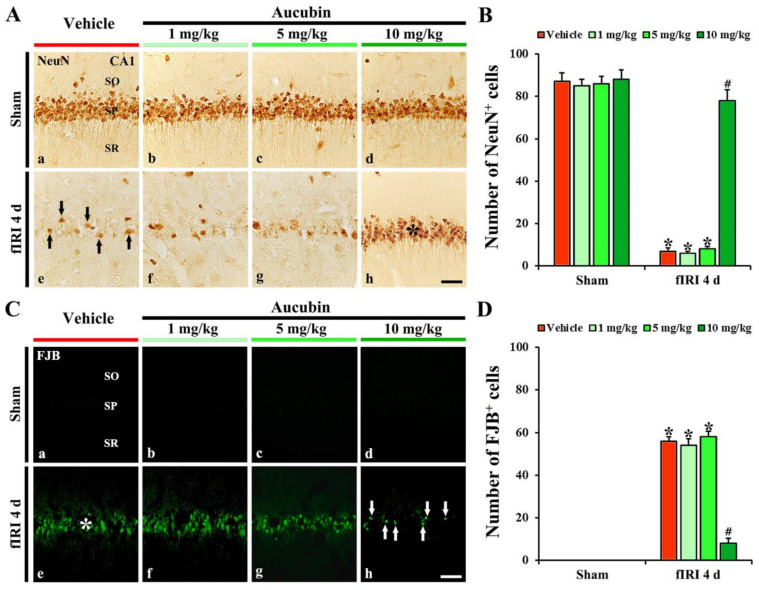
(**A**,**C**) Representative images of NeuN immunohistochemistry (**A**) and FJB fluorescence staining (**C**) in the CA1 area of the vehicle-sham (**A**(a),**C**(a)), vehicle-fIRI (**A**(e),**C**(e)), 1 mg/kg aucubin-sham (**A**(b),**C**(b)), 1 mg/kg aucubin-fIRI (**A**(f),**C**(f)), 5 mg/kg aucubin-sham (**A**(c),**C**(c)), 5 mg/kg aucubin-fIRI (**A**(g),**C**(g)), 10 mg/kg aucubin-sham (**A**(d),**C**(d)), and 10 mg/kg aucubin-fIRI (**A**(h),**C**(h)) groups at 4 days after fIRI. In the vehicle-fIRI group, a few NeuN^+^ (black arrows in (**A**(e))) and many FJB^+^ (white asterisk in (**C**(e))) pyramidal neurons in the stratum pyramidale (SP) are shown, whereas, in the 10 mg/kg aucubin-fIRI group, abundant NeuN^+^ (black asterisk in (**A**(h))) and a few FJB^+^ (white arrows in (**C**(h))) pyramidal neurons are found in the SP. SO, stratum oriens; SR, stratum radiatum. Scale bar = 60 µm. (**B**,**D**) The mean numbers of NeuN^+^ (**B**) and FJB^+^ (**D**) pyramidal neurons in the CA1 area. Values are expressed in mean ± SEM (*n* = 7, respectively; * *p* < 0.05 vs. each sham group, # *p* < 0.05 vs. vehicle-fIRI group).

In the vehicle-sham group, strong NeuN immunoreactivity was easily distinguished in CA1 pyramidal neurons (Figure 4A(a)). In the 1, 5, and 10 mg/kg aucubin-sham groups, the immunoreactivity and number of NeuN-immunostained (NeuN^+^) CA1 pyramidal cells were not different from that shown in the vehicle-sham group (Figure 4A(b–d),B).

In the vehicle-fIRI group, a considerable decrease in the numbers of NeuN^+^ CA1 pyramidal neurons was found at 4 days after fIRI (Figure 4A(e)): the number of NeuN^+^ CA1 pyramidal neurons was 7 cells/300 µm^2^ (Figure 4B). In the 1 and 5 mg/kg aucubin-fIRI groups, the numbers of NeuN^+^ CA1 pyramidal neurons were very similar to those examined in the vehicle-fIRI group (Figure 4A(f,g),B). However, in the 10 mg/kg aucubin-fIRI group, the number of NeuN^+^ CA1 pyramidal neurons (79 cells/300 µm^2^) was significantly preserved when compared to that obtained from the vehicle-fIRI group (Figure 4A(h),B).

#### 3.2.3. Findings by FJB Histofluorescence

In all sham groups, no FJB histofluorescence was observed in any cells in the CA1 area (Figure 4C(a–d)). However, in the vehicle-fIRI group, many FJB^+^ CA1 pyramidal neurons (57 cells/300 µm^2^) were detected at 4 days after fIRI (Figure 4C(e),D). In addition, in the 1 and 5 mg/kg aucubin-fIRI groups, the numbers of FJB^+^ CA1 pyramidal neurons were similar to those obtained from the vehicle-fIRI group (Figure 4C(f,g),D). However, in the 10 mg/kg aucubin-fIRI group, a few FJB^+^ CA1 pyramidal neurons (7 cells/300 µm^2^) were detected when compared to those shown in the vehicle-fIRI group (Figure 4C(h),D).

Based on the results of Nissl staining, NeuN immunohistochemistry, and FJB fluorescence staining, 10 mg/kg of aucubin showed clear neuroprotection. Therefore, 10 mg/kg of aucubin was used to examine the neuroprotective mechanisms of aucubin as follows.

### 3.3. Attenuation of fIRI-Induced Oxidative Stress by Aucubin

#### 3.3.1. Findings by DHE Fluorescence

**Figure 5:**
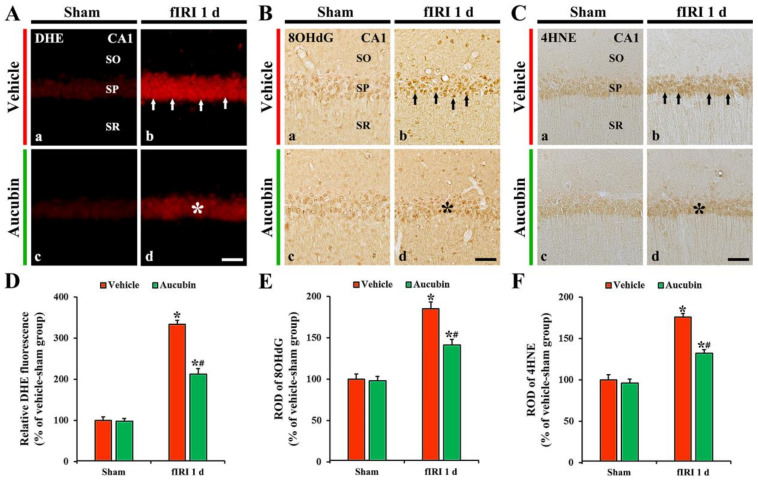
(**A**–**C**) Representative images of DHE fluorescence staining (**A**), 8OHdG (**B**), and 4HNE (**C**) immunohistochemistry in the CA1 area of the vehicle-sham (**A**(a),**B**(a),**C**(a)), vehicle-fIRI (**A**(b),**B**(b),**C**(b)), aucubin-sham (**A**(c),**B**(c),**C**(c)), and aucubin-fIRI (**A**(d),**B**(d),**C**(d)) groups 1 day after fIRI. In the vehicle-fIRI group, DHE fluorescence, 8OHdG, and 4HNE immunoreactivity in CA1 pyramidal neurons (arrows in (**A**(b),**B**(b),**C**(b))) are dramatically increased. However, in the aucubin-fIRI group, DHE fluorescence, 8OHdG, and 4HNE immunoreactivity are significantly lower (asterisks in (**A**(d),**B**(d),**C**(d))) than that shown in the vehicle-fIRI group. SO, stratum oriens; SP, stratum pyramidale; SR, stratum radiatum. Scale bar = 60 µm. (**D**–**F**) Quantitative analyses of DHE fluorescence intensity (**D**), 8OHdG (**E**), and 4HNE (**F**) in CA1 pyramidal neurons. Values are expressed in mean ± SEM (*n* = 7, respectively; * *p* < 0.05 vs. each sham group, # *p* < 0.05 vs. vehicle-fIRI group).

In all sham groups, no DHE fluorescence was evident in CA1 pyramidal neurons (Figure 5A(a,c)). In the vehicle-fIRI group, the intensity of DHE fluorescence in the CA1 pyramidal neurons was dramatically increased (approximately 334% of the vehicle-sham group) 1 day after fIRI (Figure 5A(b),D). However, in the aucubin-fIRI group, DHE fluorescence intensity was significantly lower (approximately 63%) than that evaluated in the vehicle-fIRI group (Figure 5A(d),D).

#### 3.3.2. Findings by 8OHdG Immunohistochemistry

In all sham groups, 8OHdG immunoreactivity was weakly observed in CA1 pyramidal neurons (Figure 5B(a,c),E). In the vehicle-fIRI group, 8OHdG immunoreactivity was significantly increased in the CA1 pyramidal neurons 1 day after fIRI by approximately 186% when compared with that shown in the vehicle-sham group (Figure 5B(b),E). However, in the aucubin-fIRI group, 8OHdG immunoreactivity in the CA1 pyramidal neurons was significantly low (approximately 76%) when compared to that observed in the vehicle-fIRI group (Figure 5B(d),E).

#### 3.3.3. Findings by 4HNE Immunohistochemistry

In all sham groups, 4HNE immunoreactivity was weakly observed in the CA1 pyramidal neurons (Figure 5C(a,c)). In the vehicle-fIRI group, 4HNE immunoreactivity in the CA1 pyramidal neurons was significantly increased 1 day after fIRI by approximately 175% when compared with that observed in the vehicle-sham group (Figure 5C(b),F). However, in the aucubin-fIRI group, 4HNE immunoreactivity in the CA1 pyramidal neurons was significantly lower (approximately 75%) than that shown in the vehicle- fIRI group (Figure 5C(d),F).

### 3.4. Increase of Endogenous Antioxidant Enzymes by Aucubin

#### 3.4.1. Findings by SOD1 Immunohistochemistry

**Figure 6:**
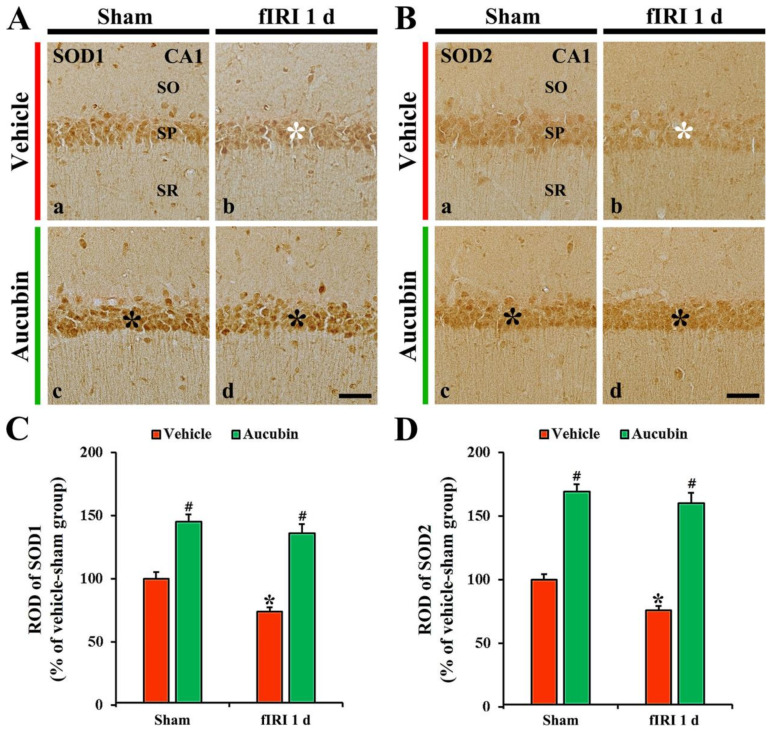
(**A**,**B**) Representative images of SOD1 (**A**) and SOD2 (**B**) immunohistochemistry in the CA1 area of the vehicle-sham (**A**(a),**B**(a)), vehicle-fIRI (**A**(b),**B**(b)), aucubin-sham (**A**(c),**B**(c)), and aucubin-fIRI (**A**(d),**B**(d)) groups 1 day after fIRI. In the vehicle-fIRI group, SOD1 and SOD2 immunoreactivity in pyramidal cells (white asterisks in (**A**(b),**B**(b)) are significantly reduced when compared with those found in the vehicle-sham group. In the aucubin-sham and aucubin-fIRI groups, SOD1 and SOD2 immunoreactivity in the pyramidal neurons (black asterisks in (**A**(c,d),**B**(c,d))) are apparently higher than those shown in the vehicle-sham groups. SO, stratum oriens; SP, stratum pyramidale; SR, stratum radiatum. Scale bar = 60 µm. (**C**,**D**) Quantitative analyses of SOD1 (**C**) and SOD2 (**D**) immunoreactivity in CA1 pyramidal neurons. Values are expressed in mean ± SEM (*n* = 7, respectively; * *p* < 0.05 vs. each sham group, # *p* < 0.05 vs. vehicle-fIRI group).

SOD1 immunoreactivity, in the vehicle-sham group, was principally shown in CA1 pyramidal neurons (Figure 6A(a)). In the vehicle-fIRI group, SOD1 immunoreactivity in the CA1 pyramidal neurons was significantly reduced 1 day after fIRI by approximately 74% when compared with that found in the vehicle-sham group (Figure 6A(b),C). In the aucubin-sham group, SOD1 immunoreactivity in the CA1 pyramidal neurons was significantly enhanced (approximately 146%) when compared with that observed in the vehicle-sham group (Figure 6A(c),C). In the aucubin-fIRI group, SOD1 immunoreactivity in the CA1 pyramidal neurons was not significantly altered when compared with that found in the aucubin-sham group (Figure 6A(d),C).

#### 3.4.2. Findings by SOD2 Immunohistochemistry

In the vehicle-sham group, SOD2 immunoreactivity was principally shown in CA1 pyramidal neurons (Figure 6B(a)). SOD2 immunoreactivity in the CA1 pyramidal neurons, in the vehicle-fIRI group, was markedly decreased (approximately 76% of the vehicle-sham group) 1 day after fIRI (Figure 6B(b),D). In the aucubin-sham group, SOD2 immunoreactivity in the CA1 pyramidal cells was significantly enhanced (approximately 170%) when compared with that found in the vehicle-sham group (Figure 6B(c),D). SOD2 immunoreactivity in the CA1 pyramidal neuron cells, in the aucubin-fIRI group, was similar to that observed in the aucubin-sham group (Figure 6B(d),D).

### 3.5. Increase of Neurotrophic Factor Protein Levels by Aucubin

**Figure 7:**
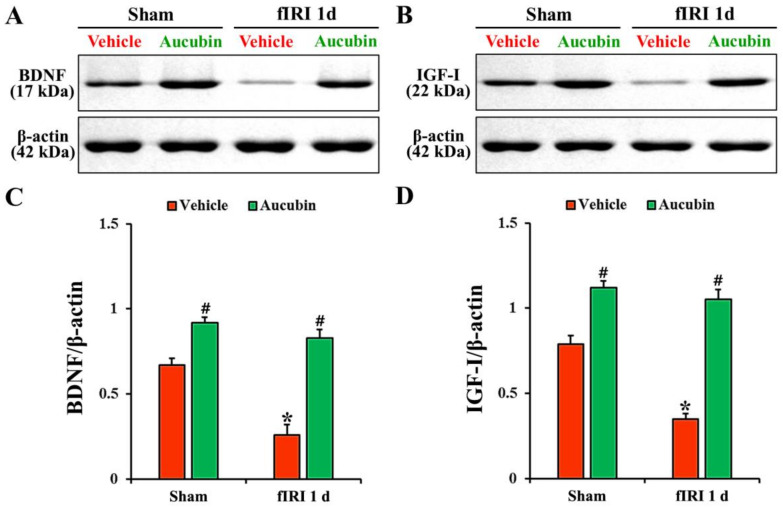
(**A**,**B**) Representative Western blot images of BDNF and IGF-I in the CA1 area of the vehicle-sham, vehicle-fIRI, aucubin-sham, and aucubin-fIRI groups 1 day after fIRI. In the vehicle-fIRI group, BDNF and IGF-I levels are significantly reduced when compared with those found in the vehicle-sham groups. In the aucubin-sham and aucubin-fIRI groups, BDNF and IGF-I levels are significantly higher than those observed in the vehicle-sham groups. (**C**,**D**) Quantitative analyses of BDNF and IGF-I levels by normalization to β-actin level, respectively. Values are expressed in mean ± SEM (*n* = 5, respectively; * *p* < 0.05 vs. each sham group, # *p* < 0.05 vs. vehicle-fIRI group).

In the vehicle-sham group, BDNF and IGF-I proteins were detected in the CA1 area by Western blot (Figure 7A,B). The levels of BDNF and IGF-I, in the vehicle-fIRI group, were significantly decreased 1 day after fIRI by approximately 61% and 56%, respectively, when compared with those observed in the vehicle-sham group (Figure 7A–D). In the aucubin-sham group, the levels of BDNF and IGF-I were significantly higher (approximately 14-fold and 1.5-fold, respectively) than those revealed in the vehicle-sham group (Figure 7A–D). The levels of BDNF and IGF-I in the aucubin-fIRI group were not significantly different from those shown in the aucubin-sham group (Figure 7A–D).

## 4. Discussion

Natural constituents derived from medicinal plants have been perceived as promising candidates for use in future clinical applications in cerebral ischemia due to their various pharmacological actions and safety aspects [11,31,32]. It has been reported that oleuropein, which is a glycosylated seco-iridoid found in green olive skin, flesh, seeds, and leaves [33], offers a neuroprotective effect against ischemic stroke through its antioxidative and antithrombotic activities in a rat model of ischemic stroke [34]. Here, we first evaluated the neuroprotective effects of aucubin, as an iridoid glycoside, in gerbils subjected to 5-min transient ischemia in the forebrain which produces massive pyramidal neuron death in the hippocampal CA1 area from 4 days after fIRI. In addition, short-term memory function evaluated on day 4 after fIRI was apparently kept in the 10 mg/kg aucubin-fIRI group, not in the 1 and 5 mg/kg aucubin-fIRI groups. Furthermore, our current results of Nissl staining, NeuN immunohistochemistry, and FJB fluorescence staining showed that pretreatment with 10 mg/kg, but not 1 or 5 mg/kg, of aucubin protected hippocampal CA1 pyramidal neurons from fIRI, indicating that the dose of 10 mg/kg aucubin can display neuroprotective effects against fIRI. This finding is the first regarding the neuroprotective potentiality of aucubin in brain ischemic insults. Basically, neuroprotective effects are hardly accomplished without according to “dose-dependence”; and the minimum dosage exerting neuroprotective effects can be determined. If the neuroprotective effect was exerted in a dose-dependent manner, approximately half of the pyramidal neurons in the hippocampal CA1 region should survive following fIRI when 5 mg/kg aucubin was administrated. However, our present results showed that pretreatment with 5 mg/kg aucubin failed to protect pyramidal neurons from fIRI. We have reported such minimum dosages to display the neuroprotective effects of neuroprotective materials in a gerbil model of fIRI. The neuroprotective effect of Korean red pine (*Pinus densiflora*) Bark Extract (PBE) is shown when 100 mg/kg PBE, but not 25 or 50 mg/kg PBE, is pretreated [35]. In addition, chlorogenic acid (CGA), an ester of caffeic acid and quinic acid, exerts neuroprotection when 30 mg/kg CGA, but not 7.5 or 15 mg/kg CGA, is administrated [36]. Taken together, neuroprotective effects by pretreatment with protective materials in animal models of transient brain ischemia might not be displayed dose-dependently. We added this discussion in the first paragraph.

As a mechanism of the neuroprotection of aucubin against fIRI in this experiment, pretreated aucubin protected CA1 pyramidal neurons cells from fIRI by attenuating oxidative stress. It is well known that oxidative stress refers to elevated intracellular levels of ROS. In this study, treatment with 10 mg/kg of aucubin in the aucubin-fIRI group significantly reduced IRI-induced superoxide anion production compared with the vehicle-fIRI group when DHE (a probe for superoxide anion) fluorescence staining was conducted, indicating that 10 mg/kg of aucubin has neuroprotective potential against brain ischemic stroke.

The formation of ROS above permissible levels following cerebral ischemia and reperfusion causes oxidative stress-related damage of major cellular constituents, such as nucleic acids, membrane lipids, and proteins, ultimately leading to neuronal death in ischemic brain tissue [29,37]. In our current study, the immunoreactivities of 8OHdG (an oxidized product of DNA) and 4HNE (a lipid peroxidation product) were significantly increased in hippocampal CA1 pyramidal neurons of the vehicle-fIRI group 1 day after fIRI: this finding is consistent with our previous studies using gerbils [29,38]. However, in the aucubin-fIRI group, the increased immunoreactivities of 8OHdG and 4HNE were dramatically attenuated 1 day after fIRI. Kim et al. (2020) have reported that pretreated indole-3-propionic acid (IPA), one of the tryptophan-derived indole compounds, saves hippocampal CA1 pyramidal neurons from fIRI in gerbils, showing that increased 8OHdG immunoreactivity in the ischemic CA1 pyramidal neurons is significantly attenuated by IPA pretreatment. In addition, it has been reported that the pretreatment of laminarin, a polysaccharide isolated from brown algae, effectively protects the CA1 pyramidal neurons from fIRI in gerbils, showing that 4HNE expression is significantly decreased in the ischemic CA1 pyramidal neurons (Park et al., 2020b). Taken together, pretreatments with antioxidants, such as aucubin, IPA, and laminarin, can effectively save neurons from ischemic brain injury.

Endogenous antioxidant enzymes possess ROS scavenging ability and play a crucial role in protecting neurons from oxidative damage induced by cerebral ischemia and reperfusion [39,40]. Among endogenous antioxidant enzymes, SODs have been considered as a promising target for the prevention and treatment of ischemic brain injury, because SODs form the first line of defense against ROS-mediated damage following ischemic insults. In this regard, experimental animal studies have shown that the overexpression of SOD1 in transgenic rats reduces oxidative stress and provides neuroprotection in the hippocampal CA1 area after global cerebral ischemia and reperfusion [41,42]. Similarly, the overexpression of SOD2 in transgenic mice produces neuroprotection from oxidative damage following transient focal cerebral ischemia and reperfusion [43]. In our current study, we found that the immunoreactivities of SOD1 and SOD2 were significantly decreased in hippocampal CA1 pyramidal neurons of the vehicle-fIRI group 1 day after fIRI, which is consistent with our previous studies [29,38]; however, SOD1 and SOD2 immunoreactivities were significantly increased in the aucubin-sham and aucubin-fIRI groups. These results indicate that aucubin has potent antioxidant activities against fIRI-induced oxidative damage, which may be closely related to the neuroprotection of aucubin against fIRI.

Finally, in addition to the antioxidant enzymes, neurotrophic factors, such as BDNF and IGF-I, are also potential candidates for defense against ischemic brain injury. The results of our current study revealed that the protein expression levels of BDNF and IGF-I in ischemic hippocampal CA1 area were significantly decreased in the vehicle-fIRI group 1 day after fIRI; however, the expression levels of BDNF and IGF-I in the aucubin-fIRI group were significantly higher than that in the vehicle-fIRI group, showing that the BDNF and IGF-I expression levels in the aucubin-sham group were apparently higher than those shown in the vehicle-sham group. In this connection, studies have shown that the exogenous administration of BDNF or IGF-I effectively protects hippocampal CA1 pyramidal neurons from transient global brain ischemic injury in rats [44,45]. Additionally, Park et al. (2014) have demonstrated that pretreatment with tanshinone I, a medicinal plant-derived component, protects CA1 pyramidal neurons from fIRI in gerbils, showing that the expressions of endogenous BDNF and IGF-I as well as antioxidant enzymes are elevated in the ischemic hippocampal CA1 area. Taken together, it seems that the increase of BDNF and IGF-I in the hippocampal CA1 area by aucubin pretreatment contributes to neuroprotection against fIRI.

## 5. Conclusions

In conclusion, our study revealed that pretreated 10 mg/kg of aucubin protected hippocampal CA1 pyramidal neurons, which are principal cells, from fIRI in a gerbil model of transient forebrain ischemia. The proposed mechanism of the protection involved the suppression of oxidative stress, showing that aucubin pretreatment significantly reduced fIRI-induced superoxide anion production, oxidative DNA damage, and lipid peroxidation in ischemic CA1 pyramidal neurons, and significantly increased the expression levels of endogenous SOD1 and SOD2 in the ischemic CA1 pyramidal neurons. In addition, aucubin treatment significantly enhanced the expression levels of neurotrophic factors, BDNF and IGF-I, in the ischemic hippocampal CA1 area. The data obtained here indicate that aucubin may be effective in the protection of brain ischemia and reperfusion injury as an additional neuroprotective drug.

## Data Availability

The data presented in this study are available on request from the corresponding authors.
